# Personalized Cognitive Training in Unipolar and Bipolar Disorder: A Study of Cognitive Functioning

**DOI:** 10.3389/fnhum.2013.00108

**Published:** 2013-05-13

**Authors:** Marek Preiss, Evelyn Shatil, Radka Čermáková, Dominika Cimermanová, Ilana Ram

**Affiliations:** ^1^Department of Psychology, University of New York in PraguePrague, Czech Republic; ^2^Department of Psychology, Prague Psychiatric CenterPrague, Czech Republic; ^3^Department of Psychology, Centre for Psychobiological Research, Max Stern Academic College of Emek YezreelEmek Yezreel, Israel; ^4^CogniFit LtdYokneam Ilit, Israel; ^5^Filozofická Fakulta Univerzity Karlovy v PrazePrague, Czech Republic

**Keywords:** cognitive training, unipolar depressive disorder, bipolar disorder, personalized medicine, plasticity, computer assisted training

## Abstract

Patients with unipolar depressive disorder and in the depressive phase of bipolar disorder often manifest psychological distress and cognitive deficits, notably in executive control. We used computerized cognitive training in an attempt to reduce psychological affliction, improve everyday coping, and cognitive function. We asked one group of patients (intervention group) to engage in cognitive training three times a week, for 20 min each time, for eight consecutive weeks. A second group of patients (control group) received standard care only. Before the onset of training we administered to all patients self-report questionnaires of mood, mental and psychological health, and everyday coping. We also assessed executive control using a broad computerized neurocognitive battery of tests which yielded, among others, scores in Working Memory, Shifting, Inhibition, Visuomotor Vigilance, Divided Attention, Memory Span, and a Global Executive Function score. All questionnaires and tests were re-administered to the patients who adhered to the study at the end of training. When we compared the groups (between-group comparisons) on the amount of change that had taken place from baseline to post-training, we found significantly reduced depression level for the intervention group. This group also displayed significant improvements in Shifting, Divided Attention, and in the Global executive control score. Further exploration of the data showed that the cognitive improvement did not predict the improvements in mood. Single-group data (within-group comparisons) show that patients in the intervention group were reporting fewer cognitive failures, fewer dysexecutive incidents, and less difficulty in everyday coping. This group had also improved significantly on the six executive control tests and on the Global executive control score. By contrast, the control group improved only on the reports of cognitive failure and on working memory.

## Introduction

Marvel and Paradiso (2004) pinpoint three major categories of deficits as related to depressive disorders: attention, executive function, and memory. In the section on attention, they summarize it as follows:

…patients with mood disorders can experience measurable deficit of attention during euthymic and disturbed mood states. Because attention and working memory are cognitive functions that are integral to many types of neuropsychological tests, the interpretation of studies showing deficit in a broad range of cognitive abilities should take into account the role of poor attentional/working memory capacity in these patients (p. 2).

In the case of memory, they point to unimpaired abilities of depressive patients in the area of implicit memory. In their view, the impairment of attention is also supported by structural abnormalities in the dorsolateral prefrontal cortex, functional abnormality, and post-mortem evidence in the same area.

Cognitive deficits in unipolar depression are consistently found. Paelecke-Habermann et al. (2005) found a significant deficit in the area of attention and of executive functions, as compared to control participants. According to Kessing (1998), the cognitive deficit becomes more severe with every consecutive episode by on CAMCOG battery and MDRS test. According to Paradiso et al. (1997), the *z*-scores of patients with unipolar depression in remission, as compared to controls, ranged from almost one standard deviation below the average till average performance. Neurocognitive abnormalities in major depression therefore appear to reflect a complex interaction between biased emotion processing and impaired executive control (Keedwell et al., 2009). Similarly, for bipolar disorder, cognitive impairment is frequently observed during the acute and euthymic phases of the illness (Mann-Wrobel et al., 2011). It seems that there is some degree of familial clustering of both unipolar and bipolar disorder, suggesting that there may be an overlap of (genetically determined) endophenotype markers (Keedwell et al., 2009).

Paelecke-Habermann et al. (2005) advocate the use of “neurocognitive training” with depressive patients, similarly as in the case of other groups of patients, e.g., schizophrenics. The training suggested by Paelecke-Habermann et al. (2005) is an extension of Cognitive Behavioral Therapy (CBT) or antidepressant therapy that includes training executive functions and attention. They suggested this kind of treatment, or training, can be especially important for patients that were diagnosed with impairment in their pre-morbid state. A need for such training is based on the results of their own study with depressive patients during the period of remission. The actual nature/content of their training is, however, not described. Some studies have shown that improvements from cognitive training do not easily transfer to new tasks or that they transfer only to tasks with the same processing requirements as the trained tasks (Ball et al., 2002; Willis et al., 2006), with the effect sizes for a cognitive stimulation intervention being quite small (Papp et al., 2009). However, other studies have shown that cognitive improvements can transfer to new tasks, including those with different processing requirements than the trained task (Mahncke et al., 2006; Smith et al., 2009).

It is unclear which types of cognitive training programs are the most effective in improving cognitive skills (Thompson and Foth, 2005). It appears that training approaches that are designed to accommodate each individual’s neurocognitive strengths and weaknesses, as well as those that offer instant item-specific feedback and dynamically adapt the training program accordingly, are especially effective, particularly in populations with particular cognitive enhancement needs (Whitmer and Gotlib, 2012).

The cognitive training program used in the present study achieves this by: (i) using a baseline cognitive evaluation to individualize the training regimen (ii) continually adapting the difficulty level to the subject’s performance using an interactive-adaptive system (iii) providing detailed graphic and verbal feedback after each training task. This program has previously been shown to improve trained cognitive abilities (Horowitz-Kraus and Breznitz, 2009; Shatil et al., 2010; Verghese et al., 2010; Peretz et al., 2011) as well as untrained skills such as word recognition in reading (Shatil and Share, 2003; Horowitz-Kraus and Breznitz, 2009) and walking while talking (Verghese et al., 2010). In addition, these studies show that the personalization algorithms in the program might successfully identify and train population-specific deficits. Thus, from among a large set of abilities assessed, the program identified and improved the specific cognitive abilities known to decline in special populations. Working memory was enhanced in students with dyslexia (Horowitz-Kraus and Breznitz, 2009); naming, memory, attention, and speed of processing deficits in multiple sclerosis (Shatil et al., 2010); executive control and speed of processing deficits in frail elderly at risk for falls (Verghese et al., 2010); and working memory and attention deficits in normally aging older persons (Peretz et al., 2011).

Based on the body of evidence showing that deficits in attention and executive control (Marvel and Paradiso, 2004; Paelecke-Habermann et al., 2005; Kramer and Morrow, in press) are a key symptom of depression (Basso and Bornstein, 1999), we tried improving these neurocognitive functions, as well as other everyday functions in a group of patients by using this cognitive training program. Thus, the present study sought to examine the impact of cognitive training on self-reported everyday functioning, and on executive control and attention scores obtained using a multi-domain computerized neurocognitive battery of tasks in outpatients with unipolar and bipolar depression. This battery also included tasks not measuring executive control.

## Materials and Methods

### Participants

Participants were long-term outpatients attending the Clinic of Prague Psychiatric Center. Participants eligible for inclusion had an ICD-10 diagnosis of unipolar depressive disorder or depressive phase of bipolar disorder, were Czech speakers, owned and were able to use a home personal computer, and expressed an interest in taking part in the study. Exclusionary criteria included any neurological disease and drug or alcohol abuse or dependence.

### Design

Participants were assigned to the intervention group or control group using pairwise matching on diagnosis (unipolar and bipolar), gender, and age. The intervention group received both standard care and cognitive training. The control group received only standard care. Everyday functioning and neurocognitive performance were measured prior to the initiation of training and, again, at the end of training. The study was conducted at the Prague Psychiatric Center in the years 2010 and 2011. The protocol was approved by the Ethics Committee of the Prague Psychiatric Center and written informed consent was obtained from all participants prior to the baseline assessment.

#### The interventions

##### The cognitive training intervention

CogniFit, a personalized, computer-based, online cognitive training program which has been validated in several populations (Horowitz-Kraus and Breznitz, 2009; Shatil et al., 2010; Verghese et al., 2010; Peretz et al., 2011; Haimov and Shatil, 2013; Shatil, 2013) was selected for this study. The version used for the present study offered training on multiple cognitive domains. It consisted of 21 different training tasks each with three levels of difficulty (easy, moderate, and difficult). Each training session included a mixture of visual, auditory, and cross-modality tasks aimed at training a wide range of cognitive processes. Personalization of learning is accomplished by using a baseline neurocognitive evaluation, the results of which determine the individual content and level of subsequent training for each participant. During training, personalization is maintained by an adaptive feature that continually measures the subjects’ performance, adapts the difficulty level of the training tasks, and provides detailed graphic and verbal performance feedback during and after each training task. Because the training regimen is designed based on the results on the neurocognitive evaluation and because the program continually adapts to each person’s strengths and weaknesses, it is unlikely that two participants can receive the same training regimen as regards choice of training tasks, amount, and intensity of training on each cognitive domain. The intervention lasted 8 weeks. It comprised three sessions a week, each session 20 to 30 min in length. A research assistant assisted in installing the program on the participant’s home computer. To monitor adherence, participants received a telephone call every 2 weeks to inquire on their progress.

##### Standard care

Standard care at the Prague Psychiatric Center Clinic may include regular visits to a psychiatrist, medication prescription and supervision, individual or group therapy, and access to a social worker.

#### Primary outcome measures: self-report of everyday functioning and cognitive function

To ascertain the everyday ecological validity of the cognitive training intervention, we administered the following mood and cognition self-report questionnaires.

##### Cognitive failures questionnaire

This questionnaire was administered to participants and to relatives or caregivers (in intervention group). The Czech version of the Cognitive Failures Questionnaire (CFQ) (Broadbent et al., 1982) was used. The CFQ consists of 25 items measuring the frequency of everyday cognitive failures or lapses in the general population. These covered failures of memory, attention, motor function, and perception. Each item was rated for frequency in the past 6 months, from 4 (“very often”) to 0 (“never”). The maximum score is 100. Higher total CFQ scores reflected a higher frequency of self-reported cognitive failures. The CFQ-SO version for relatives with eight items was also used.

##### Dysexecutive questionnaire

This questionnaire was also administered to participants and to relatives or caregivers (in intervention group). The Dysexecutive Questionnaire (DEX) is a 20 item checklist (Moritz et al., 2004) that is rated on a 5-point frequency scale from “never” to “very often.” It measures three broad factors (cognitive, behavioral, and emotional) and asks individuals to rate the frequency of occurrence of certain dysexecutive characteristics (e.g., abstract thinking, impulsivity, confabulation, and planning problems). Parallel versions of the questionnaire were developed for a close friend or relative who gave information about the patient (DEX-SO). Higher total DEX scores reflect higher frequencies of self-reported dysexecutive failures.

##### Everyday memory questionnaire

The Everyday Memory Questionnaire (EMQ) (Sunderland et al., 1983) consists of 28 items, each describing everyday activities that may involve memory failure. The 28-item scale was developed with 22 items representing valid memory difficulties, and six items representing “floor” or bogus items, representing atypical memory difficulties as a measure of response validity. The response format was also altered from ratings of relative frequencies (“sometimes”) to absolute values (e.g., “about once a week”), with a simplified 9-point scoring system. Higher total EMQ scores reflect a higher frequency of self-reported memory failures.

##### Schwartz outcomes scale-10

The Schwartz Outcomes Scale-10 is a brief assessment device (Blais et al., 1999) designed to measure a broad domain of psychological health. The instrument represents a broad construct related to multiple aspects of psychological functioning and psychological well-being, and it can be used with diverse populations in a wide variety of clinical settings. The instrument is sensitive to changes undertaken in psychological interventions (Dragomirecka et al., 2006b). A respondent rates each item on a scale from 0 (the statement is not accepted at all) to 6 (maximally accepted statement), as it is best applied to him/her. Higher total SOS-10 scores reflect a higher satisfaction.

##### Subjective quality of life questionnaire

To obtain standard data about QOL we used as a part of the interview an adopted Czech version (Dragomirecka et al., 2006a) of the Subjective Quality of Life Questionnaire (SQUALA) (Zannotti and Pringuey, 1992).

##### Beck depression inventory-II

BDI-II (Beck et al., 1996) was used for assessment of subjectively evaluated depressive symptoms. Higher total BDI-II scores reflect subjectively perceived depressive symptomatology.

#### Secondary outcome measures

The CogniFit evaluation was administered both before and following training. This cognitive evaluation consists of three 20-min sessions that measure a wide variety of cognitive abilities. Scores on 17 abilities are assigned using weights previously derived from a factor analysis performed on normative data from a healthy population. The CogniFit evaluation has been validated (Haimov et al., 2008) in a younger population (mean age 23 years) against several major standard neuropsychological tests, including the full Cambridge Neuropsychological Test Automated Battery (CANTAB), Raven’s Standard Progressive Matrices, the Wisconsin Card Sorting Test, the Continuous Performance Test, the STROOP test, and a variety of reading tests. For the present study, relying on the previous evidence that the program’s algorithms would identify and train specific deficits, only executive control was considered. Six executive control abilities (Working Memory, Shifting, Inhibition, Visuomotor Vigilance, Divided Attention, and Auditory Memory Span) as well as a Global executive control score averaged from these six scores were of interest.

#### Statistical analyses

The SPSS 17 computer software package was used for statistical analyses. General linear models for repeated measures were used to evaluate between-group differences in eight self-reported variables and in the seven executive control variables. A separate model was established for each variable. The independent variables were group (cognitive training or control) and time (baseline or post-training) and the dependent variable was the self-report variable or the executive control variable. Paired-samples *T*-tests were used to explore changes, from baseline to post-training scores, within each group separately. To determine whether an association exists between improvements in executive control and improvements in self-report, we calculated Pearson-moment correlations between the mood improvements and the executive control improvements and we conducted hierarchical regression analyses with executive control improvements as the independent variables and the self-report improvements as the dependent variables. We used *T*-tests for independent samples to explore between-group differences at baseline in demographic and personal details.

## Results

### Adherence and personal information

A total of 61 participants were initially enrolled. Due to high workplace load and/or insufficient knowledge of the English language, 16 participants did not start the study. From the 45 participants who started the study (*N* = 24 in the training group, *N* = 21 in the control group), 16 (35.5%) participants, 9 in the training group and 5 in the control group, did not complete it because of high workplace load, changes in health status or for no clear reasons. Thus, thirty-one participants (15 in the training group and 16 in the control group) completed the study.

Table 1 indicates that subjects did not differ in demographic and personal attributes (age, education, experience in computer use, living alone, male/female proportion, anti-depressive medication being taken during the study, and comprehension of written English) at the onset of the study.

**Table 1:** **Demographic and personal attributes data for the intervention (*N* = 15) and control groups (*N* = 16)**.

	Intervention		Control		*t*-statistics	
	Mean	SD	Mean	SD	*T*	Sig
Age	42.87	13.83	45.38	14.48	−0.882	0.385
Education (10-point scale was used)	3.53	0.743	3.69	0.602	−0.637	0.529

	%		%		Chi square	Sig

Computer use (% advanced)	42.9		57.1		2.295	0.317
Live alone (% yes)	20		6.3		4.05	0.131
Gender (% female)	66.7		56.3		0.354	0.552
Antidepressive medication (% yes)	86.7		88.2		0.005	0.945
Comprehension of written English (% poor)	26.7		37.5		0.416	0.519
Participation motivation (% not motivated)	0		12.5		2.004	0.157

### Missing values

We processed and analyzed 1736 cognitive data points (31 participants × 29 variables per administration × 2 administration). Depending on the ability, the CogniFit evaluation derives a score by combining between 4 to 10 variables. About 35 among the 1736 data points (2.01 percent) had missing data, 5 missing data points at baseline and none at post-training for the cognitive training group and 19 at baseline and 11 at post-training for the control group. Table 5 shows 4 data point had missing reaction time data measured in milliseconds and 31 data points had missing performance accuracy data measured in percentages, summed raw scores or raw scores differences. To prevent substantial loss of data from the data analysis owing to these missing data, the scores for the missing variables were “plugged” by deriving a predicted score based on an individual’s scores on the non-missing measures in the cognitive ability block. (See Cohen and Cohen, 1975). The following steps were followed to predict missing scores. All the measures used to derive an ability score were entered in a multiple regression analysis with the dependent variable being the missing variable. Predicted scores were then computed by multiplying each variable by its Beta weight and then adding up the products plus the constant. The 34 missing values were then replaced by their corresponding predicted scores.

### Primary outcomes: Self-report of everyday functioning

Table 2 shows that, except for BDI, subjects did not differ in self-report characteristics at the beginning of the study.

**Table 2:** **Means and standard deviations for the self-reported methods at the onset (PRE) of the study**.

	CogniFit group			Control group			*t*-Statistics	
	*N*	Mean	Std. deviation	*N*	Mean	Std. deviation	*T*	Sig (two-tailed)*
BDI	15	14.27	12.44	16	6.19	4.13	2.395	0.028
CFQ	15	60.87	15.27	16	57.50	14.04	0.640	0.527
CFQ-SO	15	15.35	4.43	16	13.28	4.25	1.327	0.195
DEX	15	39.53	11.21	16	32.54	10.19	1.820	0.079
DEX-SO	15	33.97	6.63	16	30.16	9.48	1.287	0.208
EMQ	15	66.00	27.75	16	54.44	21.09	1.311	0.200
SOS-10	15	36.53	13.32	16	44.31	5.88	−2.080	0.051
SQUALA	15	136.67	32.16	16	122.44	29.73	1.280	0.211

*Values smaller or equal to 0.05 are significant at 0.05 level; values smaller or equal to 0.001 are significant at the 0.001 level.

#### Between-groups comparisons

In the between-groups comparison, using the General Linear Model for Repeated Measures, the training group showed significant post-training differences as compared to the control group on BDI (Table 3). Cohen’s *d*, calculated for the self-report questionnaires, showed small-sized (CFQ; SOS-10), medium-sized (EMQ), and large-sized (BDI and DEX) effects, all in favor of the cognitive training group. These results show that the cognitive training group, but not the control group, reported clinically significant improvement in levels of depression (lower depression), in experienced cognitive failures (fewer experienced failures), experienced executive failures (fewer instances of such reported failures), experienced memory failures (fewer such reported incidents), and higher levels of satisfaction and well-being.

**Table 3:** **Between-group differences statistics for the self-report by group interactions (GLM)**.

	df	*F*	Sig. (two-tailed)*	Cohen’s d**	Cohen’s *d* effect size
BDI	1	6.113	0.020	−0.88	Large-sized effect
CFQ	1	0.718	0.404	−0.31	Small-sized effect
CFQ-SO	1	0.092	0.763	0.11	No effect
DEX	1	0.756	0.392	−1.13	Large-sized effect
DEX-SO	1	0.216	0.646	0.16	No effect
EMQ	1	3.378	0.076	−0.65	Medium-sized effect
SOS-10	1	1.724	0.199	0.47	Small-sized effect
SQUALA	1	0.370	0.548	−0.22	No effect

*Values smaller or equal to 0.05 are significant at 0.05 level.

**Cohen’s d – The minus signs indicate that the second mean difference (in this case, the control group’s mean difference) was larger than the first mean difference (the cognitive training mean). Such higher mean differences reflect higher levels of depression, and more incidents of cognitive, memory, and executive failure. Cohen’s d interpretation: 0.2–0.49 = small effect; 0.5–0.79 = medium effect; 0.8 and higher = large effect.

#### Within-group differences

Tables 4 which measure within-group change, indicate that improvement occurred mostly for the cognitive training group. For this group, there were significant changes in four out of eight variables – BDI-II, CFQ, DEX, and EMQ. For the control group, there was a significant change in CFQ, but not in other variables.

**Table 4:** **Within-group differences for the (A) training group and (B) control group on the self-report variables, before and after training**.

	Baseline			Post-training			*t*-Statistics		
	*N*	Mean	Std. deviation	*N*	Mean	Std. deviation	*T*	df	Sig. (two-tailed)*
**(A) COGNIFIT GROUP**									
BDI	15	14.27	12.435	15	8.33	9.447	2.806	14	0.014
CFQ	15	60.87	15.268	15	53.33	13.584	3.697	14	0.002
CFQ-SO	15	15.35	4.428	15	14.96	5.534	0.275	14	0.787
DEX	15	39.53	11.211	15	34.20	9.944	2.411	14	0.030
DEX-SO	15	33.97	6.634	15	35.50	14.578	−0.439	14	0.668
EMQ	15	66.00	27.749	15	50.20	20.104	2.639	14	0.019
SOS-10	15	36.53	13.320	15	40.47	13.087	−1.891	14	0.080
SQUALA	15	136.67	32.162	15	128.60	32.997	1.125	14	0.280
**(B) CONTROL GROUP**									
BDI	16	6.19	4.135	16	5.88	4.731	0.325	15	0.749
CFQ	16	57.50	14.038	16	52.38	11.278	2.587	15	0.021
CFQ-SO	16	13.28	4.247	16	12.38	1.875	0.912	15	0.376
DEX	16	32.54	10.189	16	29.50	8.287	2.029	15	0.061
DEX-SO	16	30.16	9.483	16	30.00	6.961	0.122	15	0.904
EMQ	16	54.44	21.090	16	50.31	19.172	1.633	15	0.123
SOS-10	16	44.31	5.885	16	44.19	9.887	0.055	15	0.957
SQUALA	16	122.44	29.728	16	121.50	32.737	0.102	15	0.920

*Values smaller or equal to 0.05 are significant at 0.05 level; values smaller or equal to 0.001 are significant at the 0.001 level.

### Secondary outcome: The objective measurement of executive control

Executive control scores were derived from 28 standardized (*z*-scores) variables calculated from the data generated from 10 tasks. Table 5 presents the raw, un-standardized, and unplugged baseline and post-training means on those 28 variables for each group and Table 5 shows the standardized means and standard deviations for the six executive control scores at the beginning of the study. One observes that except for auditory memory span, subjects in the training and control groups were well matched on the six executive control scores at the onset of the study.

**Table 5:** **(A) Unplugged un-standardized baseline and post-training means and standard deviations for the 33 variables in the six executive control abilities. (B) Means in *z*-scores and standard deviations for the executive control scores at the onset of the study**.

**A**									
All executive control variables	CogniFit group				Control group				
	*N*		Missing	Mean	SD	*N*	Missing	SD	SD
+ACC1_WORD_CHANGE021.1	15		0	51.38	20.74	15	1	55.69	18.40
ACC1_WORD_CHANGE021.2	15		0	57.87	14.25	15	1	49.30	17.90
+ACC1_SPACEBAR021.1	14		1	36.45	21.62	15	1	48.06	28.78
ACC1_SPACEBAR021.2	15		0	56.92	21.49	15	1	49.44	25.69
+ACC_ALL_063.1	15		0	94.17	9.42	16	0	96.87	3.23
ACC_ALL_063.2	15		0	96.11	6.95	16	0	95.83	6.97
−DIST_TOTAL_SEGSUM020.1	15		0	9.91	7.90	15	1	10.80	9.95
DIST_TOTAL_SEGSUM020.2	15		0	6.95	1.79	16	0	7.39	2.66
−DIST_TOTAL021.1	15		0	22.71	28.24	15	1	13.40	6.00
DIST_TOTAL021.2	15		0	10.86	2.43	15	1	12.46	5.19
−DIST_TOTAL028.1	15		0	21.76	25.27	15	1	11.75	3.53
DIST_TOTAL028.2	15		0	16.41	28.31	15	1	27.79	63.92
+ACC1_028.1	15		0	58.74	20.12	15	1	65.36	11.66
ACC1_028.2	15		0	72.43	14.97	15	1	65.51	18.13
+ACC1_SEGSUM020.1	15		0	82.38	14.55	15	1	84.52	13.98
ACC1_SEGSUM020.2	15		0	88.49	6.23	16	0	87.56	8.19
+ACC1_TRIALSUM020.1	15		0	69.56	7.23	15	1	66.82	15.19
ACC1_TRIALSUM020.2	15		0	77.94	6.84	16	0	71.41	7.79
+ACC021.1	15		0	93.88	13.45	15	1	97.92	3.06
ACC021.2	15		0	98.89	3.11	15	1	99.11	3.44
+ACC029.1	15		0	92.92	17.50	15	1	97.50	5.69
ACC029.2	15		0	98.33	4.99	15	1	98.33	3.71
−RT_DIFF2_063.1	15		0	−103.47	71.62	16	0	−86.22	179.24
RT_DIFF2_063.2	15		0	−96.92	129.89	16	0	−72.14	204.42
−RT_DIFF4_063.1	15		0	−217.22	154.86	16	0	−284.53	186.86
RT_DIFF4_063.2	15		0	−252.86	287.39	16	0	−198.22	158.48
−RT_CRCT_063.1	15		0	971.50	183.58	16	0	1061.06	238.76
RT_CRCT_063.2	15		0	960.07	244.86	16	0	955.37	187.43
−RTCORRECT021.1	14		1	974.15	196.40	15	1	971.74	230.05
RTCORRECT021.2	15		0	96.78	142.48	15	1	946.30	221.57
+DELTA_TRACKING021.1	15		0	6.81	13.17	15	1	5.23	10.89
DELTA_TRACKING021.2	15		0	7.94	9.98	15	1	5.62	15.69
+AVG_N_18D.1	15		0	6.07	1.49	14	1	7.50	1.83
AVG_N_18D.2	15		0	6.80	1.47	16	0	7.13	1.96
+ACC_SER_18D.1	15		0	64.39	11.01	15	1	68.17	22.37
ACC_SER_18D.2	15		0	68.67	10.68	16	0	72.16	13.63
+ACC_IT_18D.1	15		0	76.75	18.19	15	1	83.84	14.43
ACC_IT_18D.2	15		0	86.85	6.22	16	0	89.64	7.88
−RTALL060.1	15		0	2540.02	418.53	16	0	2604.67	738.36
RTALL060.2	15		0	2183.18	436.33	16	0	2250.80	666.82
−RTCORRECT060.1	15		0	2496.87	410.30	16	0	2580.78	767.65
RTCORRECT060.2	15		0	2132.15	437.59	16	0	2209.64	678.87
+ACC060.1	15		0	81.11	11.85	16	0	78.02	15.14
ACC060.2	15		0	86.22	7.80	16	0	84.90	9.24
+ACC22A.1	15		0	73.89	14.73	15	1	82.22	11.30
ACC22A.2	15		0	77.78	15.00	15	1	81.67	14.16
+ACC_SPOKEN060.1	15		0	82.44	10.87	16	0	76.67	11.55
ACC_SPOKEN060.2	15		0	83.33	10.00	16	0	81.46	10.68
+ACC_BTN2_060.1	15		0	58.79	25.91	16	0	60.80	25.00
ACC_BTN2_060.2	15		0	69.09	24.49	16	0	68.18	21.51
+ACC_BTN3_060.1	15		0	80.67	12.80	16	0	73.75	16.68
ACC_BTN3_060.2	15		0	82.67	12.23	16	0	81.25	10.88
+AVG_N_18C.1	12		3	4.96	1.20	13	1	5.42	1.27
AVG_N_18C.2	15		0	5.70	1.00	16	0	5.66	1.23
−SHIFTING_TIME20.1	15		0	339.61	46.94	15	1	357.90	84.49
SHIFTING_TIME20.2	15		0	362.51	67.74	16	0	344.48	58.93
+MAX1_18D.1	15		0	6.07	1.49	14	2	7.50	1.83
MAX1_18D.2	15		0	6.80	1.47	16	0	7.13	1.96

**B**									
	**CogniFit group**			**Control group**			***t*-Statistics**		
	***N***	**Mean**	**Std. deviation**	***N***	**Mean**	**Std. deviation**	***T***	**df**	Sig. (two-tailed)*

Global executive control score	15	−0.46	1.06	16	−0.05	0.65	−1.34	29	0.19
Working memory	15	−0.78	0.84	16	−0.58	1.03	−0.60	29	0.55
Shifting	15	−0.59	1.73	16	0.04	0.92	−1.27	29	0.21
Inhibition	15	−0.51	1.32	16	−0.21	0.78	−0.774	29	0.445
Visuomotor vigilance	15	−0.13	0.77	16	−0.16	0.98	0.10	29	0.920
Dividend attention	15	−0.45	1.44	16	0.13	0.93	−1.34	29	0.10
Auditory memory span	15	−0.34	1.30	16	0.50	1.87	−1.44	29	0.16

*Values smaller or equal to 0.05 are significant at 0.05 level; values smaller or equal to 0.001 are significant at the 0.001 level.

#### Between-groups comparisons

The between-groups comparisons (Table 6) revealed that, when compared to the control group, the training group showed significant improvements on the Global executive control score as well as on two executive control measures: shifting and divided attention. Table 6 also shows that, Working Memory, Auditory Memory Span, and Inhibition, but not Visuomotor Vigilance, manifest some clinical significance (Cohen’s *d* for these variables are of small-sized) but do not reach statistical significance.

**Table 6:** **Between-group differences statistics for the executive control by group interactions**.

	Repeated measurements statistics			Cohen’s *d*	
	df	*F*	Sig. (two-tailed)*	*d*	Cohen’s *d* effect size
Global executive control score	1	6.529	0.016	0.910	Large-sized effect
Working memory	1	1.089	0.305	0.374	Small-sized effect
Shifting	1	6.139	0.019	0.886	Large-sized effect
Inhibition	1	0.544	0.467	0.263	Small-sized effect
Visuomotor vigilance	1	0.279	0.602	0.157	No effect
Divided attention	1	5.736	0.023	0.938	Large-sized effect
Auditory memory span	1	2.543	0.122	0.406	Small-sized effect

*Values smaller or equal to 0.05 are significant at 0.05 level.; values smaller or equal to 0.001 are significant at the 0.001 level.

*Cohen’s *d* interpretation: 0.2–0.49 = small effect; 0.5–0.79 = medium effect; 0.8 and higher = large effect*.

#### Within-group differences

Change from baseline to post-training was explored, within each group separately, for the six executive abilities (Working Memory, Shifting, Inhibition, Visuomotor Vigilance, Divided Attention, and Auditory Memory Span) as well as for the Global executive control score, averaged from those six variables. Tables 7 show that subjects in the cognitive training group significantly improved on Divided Attention, working memory, and on the Global executive control score. Other cognitive abilities display trends for statistical significance. The control group improved in working memory.

**Table 7:** **Within-group differences for the (A) training group (B) control group on the executive control variables, before and after training**.

	Baseline			Post-training			*t*-Statistics		
	*N*	Mean	Std. deviation	Mean	*N*	Std. deviation	*T*	df	Sig. (two-tailed)
**(A) COGNIFIT GROUP**									
Global executive control score	15	−0.46	1.06	15	0.17	0.43	−3.43	14	0.004
Working memory	15	−0.78	0.84	15	−0.05	0.64	−4.17	14	0.001
Shifting	15	−0.59	1.73	15	0.13	0.81	−2.07	14	0.057
Inhibition	15	−0.51	1.32	15	0.00	0.88	−2.06	14	0.058
Visuomotor vigilance	15	−0.13	0.77	15	0.34	0.49	−2.05	14	0.059
Dividend attention	15	−0.45	1.44	15	0.26	0.60	−2.31	14	0.037
Auditory memory span	15	−0.34	1.30	15	0.37	0.84	−1.97	14	0.069
**(B) CONTROL GROUP**									
Global executive control score	16	−0.05	0.65	16	0.06	0.64	−1.07	15	0.299
Working memory	16	−0.58	1.03	16	−0.08	0.93	−3.41	15	0.004
Shifting	16	0.04	0.92	16	−0.26	1.18	1.31	15	0.211
Inhibition	16	−0.21	0.78	16	0.06	0.86	−1.16	15	0.264
Visuomotor vigilance	16	−0.16	0.98	16	0.15	0.64	−1.20	15	0.248
Dividend attention	16	0.13	0.93	16	−0.17	1.20	1.26	15	0.228
Auditory memory span	16	0.50	1.87	16	0.68	1.19	−0.62	15	0.547

### Does the cognitive improvement predict the improved self-report?

To answer the question of whether the self-reported improvements in everyday memory and in mood are associated with the cognitive improvement in executive control, we correlated the improved self-report mean difference (post-intervention mean minus baseline mean for BDI) with the seven executive control mean differences. None of the correlations reached statistical significance. Not unexpectedly, further efforts at uncovering a relation between self-report improvements and executive control improvements, by conducting hierarchical regressions with the self-report score as a dependent variable and the executive control scores as independent variables, were futile.

## Discussion

Fifty two percent of the participants completed the project without any extrinsic reward, with only a monitoring telephone call every 2 weeks to inquire about their progress. We consider this adherence rate satisfactory and suggestive of plausibility for future use of computer training for depressive patients.

When compared to the control participants, participants in the cognitive training group reported significantly lower levels of depression on Beck’s BDI-II. To our knowledge this is the first study to report a reduction in depressive symptomatology, following cognitive training. Although there are no similar studies to guide our interpretation of the results, a reasonable explanation could be that cognitive training offers a cognitively based coping strategy, to depressed patients who are, otherwise, often characterized by a negative cognitive pattern (Biringer et al., 2009). Also, high spontaneous remission rates could be expected in these highly motivated and only mildly depressed patients. It should be left to a controlled clinical trial to define the treatment effects against other confounding effects such as spontaneous remission or unspecific enrollment into a standardized clinical treatment setting and associated increased attention.

Another improvement observed in the training group, but which did not reach statistical significance when the groups were compared, was on the DEX (measuring dysexecutive failures in cognition, behavior, and emotion as reported by a caregiver). This finding, accompanied by significant neurocognitive improvements in shifting and in divided attention, as well as in the Global Executive Function score suggests that the present cognitive training regimen can reduce dysexecutive symptoms and that the change is such that it is noticeable to close family and caregivers.

The CFQ (reported also by the caregiver), showed significant change both within the cognitive training group and within the control group. This pattern of results may suggest that the intervention itself, regardless of its nature (standard or cognitive and standard) can reduce the self-reports perceived levels of cognitive failures. However, reduction on self-report does not necessarily means reduction in actual behavior; as mentioned above, when caregivers were asked to report failures in cognition, behavior, and emotion, improvement was seen from pre- to post-intervention only in the cognitive training group, again suggesting an advantage for including cognitive training when treating affective disorders.

Differences between the groups were explored for six executive abilities as well as for the Global executive control score, averaged from those six variables. Because the training tasks do not resemble the assessment tasks, and because the control group did not receive training, it appears unlikely that the observed improvements with cognitive training result from a practice effect. From the clinical point of view, a large-sized effect was found in the Global executive control score, Divided Attention, and Shifting. It could be expected that such large effects could influence task performance in normal life.

Individuals with depressive disorders are often found deficient on executive control and most particularly on set switching or shifting (for a review see Gotlib and Joormann, 2010). Deficiencies in switching in depressed individuals have been linked, in the literature, to perseveration in negative thought (Kircanski et al., 2012) and to rumination (Whitmer and Gotlib, 2012). These authors posit that depressed individuals have difficulty disengaging from negative thoughts and that executive control deficits primarily in switching, might be closely linked to this behavior. The largest cognitive improvement effects in our executive battery were observed in Shifting, Divided Attention, and in the Global Executive Score. It would appear, then, that the personalization feature of the program had identified these specific executive control deficits in most participants and allocated significant training time on those abilities.

Our results support the feasibility of broad, multi-domain, computerized remediation approaches in depression. From previous research, we know that cognitive deficits are often specific and population-based. It, therefore, seems that broadness, the ability to identify specific cognitive deficits from among a multiplicity of existing cognitive domains, and to subsequently train those, is an advantage in cognitive training remediation. Using this program, this approach has proven relevant to gait (Verghese et al., 2010), dyslexia (Horowitz-Kraus and Breznitz, 2009), multiple sclerosis (Shatil et al., 2010), and normal aging (Peretz et al., 2011). Alternately, the results of the present study may also support a mutually benefiting view of broader cognitive training. Visuomotor vigilance, auditory memory span, and inhibition also manifested some clinical significance (Cohen’s *d* for these variables was of medium size). These trends toward improvement suggest that broad training using several different tasks could, indirectly have affected executive control.

Our results also emphasize that cognitive deficits in depression are tractable, at least temporarily, and can be relieved using cognitive training, alone or in combination with medication or with other therapies. Yet whether more extended use of personalized cognitive training could provide additional improvement, whether the gain would be maintained over time and whether these benefits would project beyond neuropsychological test performance into daily activities are questions for future research (Peretz et al., 2011).

The last point – if the benefits would project beyond computerized neuropsychological test performance into everyday functioning – was questioned by this study. There is a gap in the literature with regard to everyday functioning among persons with depression and several factors may explain this gap, including definition and measurement issues, broadness of treatment effect issues, and symptomatology variability issues (Moore et al., 2010). Our results suggest that cognitive training brings about beneficial change in function and behavior that is tangible enough to be observed by the patient and by his close relatives.

We found no correlation between cognitive improvements and everyday functioning which may be due to unknown and not measured environmental changes, changes in self-efficacy, or changes in motivation. Alternately, as stated above, cognitive training may equip the patient with better executive control, thus allowing him or her to devise better strategies to cope with known symptoms of depression, such as rumination and perseverance (Whitmer and Gotlib, 2012). Because we did not measure pharmacological compliance, it might be that unexpected (not measured) changes in medication could intervene in everyday cognition. Also, spontaneous remission or changes in health status could influence the results. Future studies should control for these conditions.

Because family members rely on the home setting and on patients’ verbal and non-verbal behavior, they might be able to provide accurate information and even to display a more objective and unbiased attitude (Burgess et al., 1998). Yet, we found no statistically significant between-group changes in the observations of significant others. It might be that the cognitive changes or the groups size are too small or that methods other than questionnaires and rating scales should be used.

Stewart et al. (2003) conducted a study to estimate the impact of depression on labor costs in the US workforce. They found that average number of productive hours lost was considerably higher for patients with major depressive disorder (MDD), followed by patients in partial remission of major depression and dysthymia. This evidence suggests that functioning may be impaired long before and long after the major depression episode (Hirchsfeld, 1998; Ormel et al., 2004). The results of our study are promising for improving cognitive functioning in outpatients, but need to be followed up with larger scale trials to establish the validity of cognitive approaches to everyday and cognitive functioning in depression. Additional training over a longer time period may be required to observe improvements in other domains. Similarly, other computerized or non-computerized cognitive remediation programs targeting attention and executive functions might, perhaps, achieve similar executive function benefits.

Although true subject randomization and the intention-to-treat principle were not adopted in this study, thus not enabling an unbiased assessment of the efficacy of cognitive training, the results suggest that personalized cognitive training using a home-based computer assisted program is associated with some improved everyday cognition in people with depressive disorder. A Czech Statistical Office community survey in 2003 reported that 30% of Czech households use a personal computer, in 2003 it was 59%. These figures are likely higher today, suggesting a huge potential audience for computerized cognitive remediation programs.

One major limitation of the study is the lack of an active comparable control group. This could be addressed in future studies with a computer program designed to be similar to the CogniFit program, but which involves tasks that do not engage high-level cognitive functioning. A second limitation is that we had only subjective data on mood, and objective data is needed as well. A third limitation is the limited information we obtained about remission status and whether a cognitive deficit was present at baseline or not.

## Conflict of Interest Statement

Evelyn Shatil and Ilana Ram are employees of CogniFit.
